# A multiphysics-based artificial neural networks model for atherosclerosis

**DOI:** 10.1016/j.heliyon.2023.e17902

**Published:** 2023-07-07

**Authors:** M. Soleimani, B. Dashtbozorg, M. Mirkhalaf, S.M. Mirkhalaf

**Affiliations:** a Institute of Continuum Mechanics, Leibniz Universität Hannover, Hannover, Germany; b Department of Surgical Oncology, Netherlands Cancer Institute, Amsterdam, the Netherlands; c School of Mechanical, Medical and Process Engineering, Queensland University of Technology, Brisbane, Australia; d Department of Physics, University of Gothenburg, Gothenburg, Sweden

**Keywords:** Atherosclerosis, Artificial neural networks, Multi-physics, Finite Element Modeling

## Abstract

Atherosclerosis is a medical condition involving the hardening and/or thickening of arteries' walls. Mathematical multi-physics models have been developed to predict the development of atherosclerosis under different conditions. However, these models are typically computationally expensive. In this study, we used machine learning techniques, particularly artificial neural networks (ANN), to enhance the computational efficiency of these models. A database of multi-physics Finite Element Method (FEM) simulations was created and used for training and validating an ANN model. The model is capable of quick and accurate prediction of atherosclerosis development. A remarkable computational gain is obtained using the ANN model compared to the original FEM simulations.

## 1 Introduction

Atherosclerosis is a pathological condition in which the arteries wall harden and thicken [1]. The complications associated with atherosclerosis account for almost 50% of death in developed countries [2]. It is in fact the disease of large elastic arteries as well as medium-sized arteries. It manifests itself in the formation of fibro-fatty plaque in the intima (the innermost layer of the artery) that leads to the creation of a bulge in the lumen, consequently narrowing down the lumen cross-section [3]. In severe cases, it may completely obstruct the lumen and result in lethal conditions.

Pathogenesis of atherosclerosis has drawn significant attention over the last decades [4], [5], [6], discussing two schools of thought: The “Inside-out”, and the relatively newer “Outside-In” [7], [8]. In the former, the root of pathology is sought in the inside of the artery. For example, the migration of the Low-density Lipoprotein (LDL) particles from the lumen bloodstream to the intima contributes to plaque formation [9]. Conversely in the latter, the atherosclerotic plaque is believed to be driven from the outside of the artery. For instance, a recent study [10] shows that the LDL particles reach the intima through the capillaries called Vasa Vasorum (VV) that originate from the artery outside and nourish the artery wall. According to this hypothesis, if they penetrate the intima due to excessive differentiation of it, they provide a duct via which the LDL particles can reach the deeper layer of the intima and accumulate. Based on another outside-in theory [11], the first author has recently developed a mathematical model [12]. According to this novel hypothesis, atherosclerotic is described as the inflammatory response of artery walls due to the disruption of VV function. The disturbance may occur due to the occlusion of VVs with fine infectious agents such as viruses or bacteria.

Physics-based models, typically derived using continuum mechanics theory and solved by the FEM, are being increasingly used for bio-mechanics applications (see e.g. [12], [13], [14], [15]). However, more recently, to increase the computational speed of these models, machine learning techniques are being used to reduce the calculation time of the physics-based models (see e.g. [16], [17], [18]). This combination has resulted in great achievements, and highly applicable tools are developed for medical applications (see e.g. [19], [20]).

The application of artificial neural networks (ANN) is a growing trend in data analysis and has been named one of the 10 breakthrough technologies in the last decade [21]. To date, it is emerging as the leading machine-learning tool in image analysis and computer vision domains [22], [23]. ANNs are widely used for medical applications in different disciplines of health care [24]. ANN models have been extensively applied in diagnosis, treatment, and prognosis as a tool for signal processing, medical image analysis, and decision-making, see e.g., [25], [26], [27], [28]. ANNs also have been used for modeling applications in medicine and clinical research.

The main purpose of this study is to develop an accurate and computationally efficient model for atherosclerosis. For that purpose, a multi-physics model developed by Soleimani et al. [12] and artificial neural networks are used. Such a tool can serve as an assistive virtual gadget in predicting the prognosis of atherosclerosis as one of the arterial diseases. Especially, it can predict and monitor the acuteness of the artery occlusion and help the surgeon with opting for the necessary medical measures such as angioplasty. To generate the required database for training and validating an ANN model, hundreds of simulations were conducted from which, 90% of the data was used for training and the rest of the data for validation. The trained model showed a very good performance and very good comparisons to the multi-physics simulations were obtained. The main gain, however, is the computational efficiency of the ANN model compared to the original Finite Element Method (FEM) simulations. The ANN model gives predictions in less than a second, while each multi-physics FE simulation takes roughly 2 hours on the same personal computer (Quad-Core 2.66 GHz CPU and 16G RAM).

The remaining of this paper is structured as follows. Section 2 describes the multi-physics model [12] which is used for atherosclerosis analyses and generating the database for the ANN model. Section 3 illustrates the process for generating the database and gives details about the developed ANN model. Section 4 discusses the obtained results, the model accuracy, and computational performance compared to the original model. Finally, Section 5 summarizes the contribution of this study and gives an outlook for future developments.

## 2 Multiphysics mathematical model

**Figure 1:**
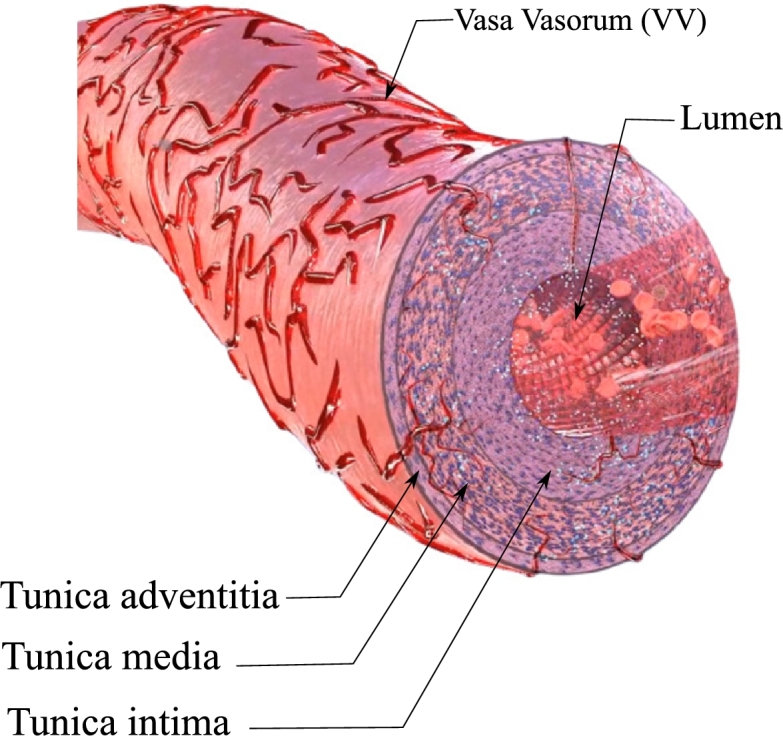
The structure of a typical elastic artery with Vasa Vasorum (VV) microvessels, from [12].

The elastic artery walls consist of three layers. Fig. 1 depicts the structure of an elastic artery as well as the VV network. While the inner regions of the wall are nourished via a diffusion mechanism from the bloodstream in the lumen, the VVs task is to provide nutrients to the outer regions of the artery wall.

The mathematical model used in this study ([12]) is based on two fundamental hypotheses:

1. The wall artery inflammation and consequently overgrowth is triggered and evolves in response to “nutrient scarcity” due to a disruption in Vasa Vasorums (VVs) function. This is based on a novel surgeon's view on atherosclerosis proposed in 2017 [11].
2. The inflammatory region spreads in the direction of the maximum nutrient gradient. This assumption is mathematical and introduced by [12] based on which the phase-field modeling of the inflammation is realized. Moreover, mechanical overgrowth is proportional to inflammation.

### 2.1 Mathematical modeling

#### 2.1.1 Governing equations

The mathematical modeling of atherosclerosis is described using a coupled multi-field approach. The mechanical deformation is represented using the vectorial variable ***u***. The scalar variable *c* captures the availability of the nutrient. Finally, the tissue inflammation (overgrowth) is represented by another scalar variable *ϕ* that is treated as a phase-field variable.

The standard and well-known equation for the balance of linear momentum reads

(1) ∇⋅σ=0,

where ***σ*** is the Cauchy stress derived based on a hyperelastic constitutive behavior [12] according to

(2) $$\sigma =\frac{1}{J_{e}}\frac{\partial \Psi }{\partial F_{e}}F_{e}^{T},$$

in which Ψ signifies the elastic free energy and $F_{e}$ designates the elastic deformation gradient and $J_{e}$ is its determinant $J_{e}=\text{Det}(F_{e})$.

The deformation gradient tensor ***F*** is multiplicatively split into two parts [12], namely elastic ($F_{e}$) and growth-related ($F_{g}$) parts according to

(3) $$F=F_{e}F_{g}.$$

It is related to the spatial gradient of the mechanical displacement ***u*** as follows

(4) $$F={\left(I-\nabla u\right)}^{-1}.$$

Here a Holzapfel–Gasser–Ogden (HGO) type anisotropic hyperelastic free energy function [29] is utilized as follows

(5) $$\Psi =\underset{\text{Matrix energy}}{\underset{︸}{\frac{\mu }{2}(I_{1e}-3)}}+\underset{\text{Collagen fiber energy}}{\underset{︸}{\frac{\eta }{\beta }e^{\beta [\rho <I_{4e}-1{>}^{2}+(1-\rho ){\left(I_{1e}-3\right)}^{2}]}}}+\underset{\text{Volumetric energy contribution}}{\underset{︸}{\frac{\nu }{\mu (1-2\nu )}{\left(J_{e}-1\right)}^{2}-\mu \text{Log}J_{e}}},$$

with $I_{1e}$, and $I_{4e}$ being the invariants of the isochoric right Cauchy-Green tensor defined as $C_{e}=J_{e}^{-\frac{2}{3}}F_{e}^{T}F_{e}$. The parameters *μ*, *ν*, *η*, *β* and *ρ* signify the material parameters.

The transport of the nutrient is assumed to obey the classical and standard diffusion equation according to

(6) $$\nabla \cdot (D\nabla c)-R_{c}=0,$$

where *c* represents the nutrient concentration and $R_{c}$ refers to the rate of nutrient consumption in the cells. Additionally, *D* denotes the diffusivity coefficient.

The inflammation of the artery is captured using a phase-field variable governed by an Allen-Cahn type [30] in accordance with

(7) $$\underset{\begin{array}{c} \text{Phase evolution}\\ \text{in pseudo time} \end{array}}{\underset{︸}{\frac{\partial \phi }{\partial t}}}=\underset{\begin{array}{c} \text{Bulk contribution} \end{array}}{\underset{︸}{-M\frac{\partial f(\phi )}{\partial \phi }}}+\underset{\begin{array}{c} \text{Sharp interface}\\ \text{contribution} \end{array}}{\underset{︸}{\epsilon^{2}\nabla^{2}\phi }}+\underset{\begin{array}{c} \text{Driver (source)}\\ \text{of phase-field} \end{array}}{\underset{︸}{S(\phi ,c)}},$$

in which the two parameters *ϵ* and *M* are two phase-field parameters controlling the sharpness of the interface between the inflammatory (ϕ=1) and the healthy (ϕ=0) tissues. The function f(ϕ) is a so-called double-well potential with the function

(8) $$f(\phi )=16M\phi^{2}{\left(1-\phi \right)}^{2}.$$

Finally, the source term S(ϕ,c) which is, in fact, the driver of the phase field is defined [12] using

(9) $$S(\phi ,c)=R_{s}\frac{\nabla \phi \cdot \nabla c}{\left|\nabla c\right|},$$

where $R_{s}$ is a parameter that controls the magnitude (intensity) of the source term. It is obvious that the coupling between the phase field and the nutrient concentration is established through the source term. Additionally, the growth part of the deformation is a direct result of inflammation and can be computed by solving the time-dependent differential equation

(10) $${\overset{˙}{F}}_{g}F_{g}^{-1}=k_{g}\overset{˙}{\phi }I,$$

in which $k_{g}$ is a parameter that controls the growth intensity. For more elaboration on the equations the readers may refer to [12].

#### 2.1.2 Boundary and initial conditions

The developed mathematical model is three-dimensional. However, the numerical examples are restricted to two-dimensional cases with plane strain assumption due to computational costs. It implies that the model is, indeed, a histological approach in which the cross-sectional data are of greater importance rather than axial ones. The total number of nodes in the 2D model amounts to $2\times 10^{4}$ leading to $8\times 10^{4}$ degrees of freedom (unknowns) due to the fact that each node has 4 degrees of freedom.^1^ To mimic the physical condition, the artery is embedded in a very soft elastic medium to account for the surrounding tissues. The impact of the pulsative blood pressure in the lumen is also considered through applying the maximum mechanical pressure on the interior surface of the artery as a conservative assumption for the alternating nature of the blood pressure.

**Figure 2:**
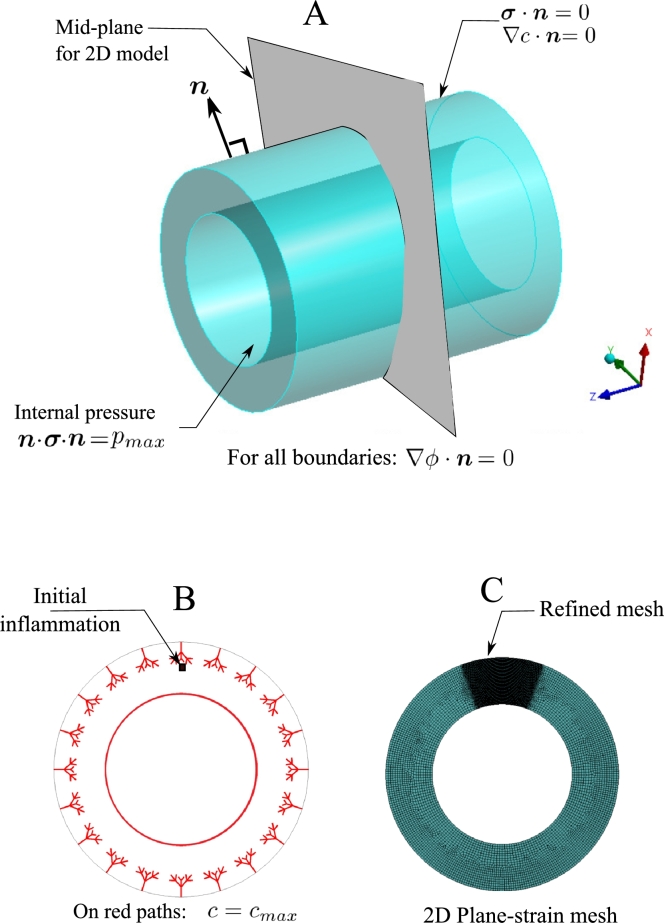
Boundary and initial conditions (A and B); Discretization details and refined zone (C).

**Figure 3:**
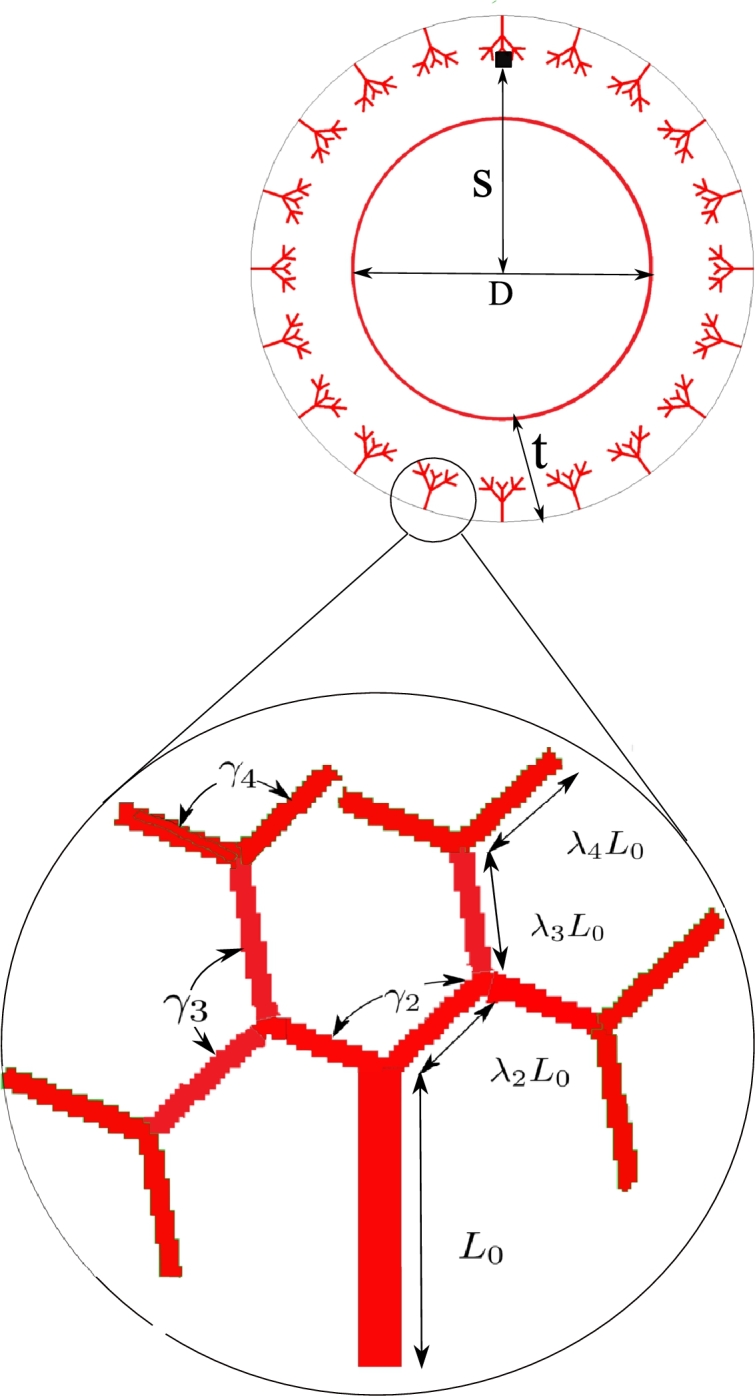
Geometrical parameters of the artery and Vasa Vasorum network.

Furthermore, the maximum value of the nutrient is prescribed on the interior surface of the artery as well as the nodes lying on the network of Vasa Vasorum. The initial perturbation is manually introduced at a certain location close to a microvessel tip. In reality, it is caused by an infectious agent, such as bacteria. This launches the nucleation of the inflammation and serves as the initial condition for the phase-field equation. Fig. 2 demonstrates the applied boundary conditions on the model. The Geometrical parameters for creating the Vasa Vasorum network are reflected in Fig. 3 and Table 1.

**Table 1:** Geometrical parameters of Vasa Vasorum tree fractal.

Description	Parameter	Value	unit
Artery inner diameter	D	50.0	μm
Artery Wall thickness	t	15.0	μm
Initial occlusion location	S	70.0	μm
Tree trunk	*L*_0_	30	μm
Second branching parameter	*λ*_2_	1.0	–
Third branching parameter	*λ*_3_	1.0	–
Forth branching parameter	*λ*_4_	1.0	–
Second branch angle 2D	*γ*_2_	$\frac{2\pi }{3}$	–
Third branch angle 2D	*γ*_3_	$\frac{2\pi }{3}$	–
Forth branch angle 2D	*γ*_4_	$\frac{2\pi }{3}$	–

### 2.2 Numerical method

To solve the set of equations introduced in the previous section, a FEM-based approach has been used. To do so, a user element has been programmed in FORTRAN to be invoked by ANSYS. Programming this user-element is inevitable due to the fact that the available elements formulation in ANSYS does not accommodate the multiphysics problem at hand. The user element is compiled and appended to the ANSYS element library. The user-element can be integrated to any FEM-Solver. Here ANSYS was adopted to benefit from its rich pre- and post-processor. The model is created in ANSYS Design Modeler. Fig. 2 shows the meshed model with a local refinement at the region of interest. More details on the numerical algorithm are available in [12]. The topology of the element is the standard brick-shaped with 8 nodes each bearing 5 degrees of freedom, namely three components of displacements (***u***), nutrient concentration (*c*), and inflammation (*ϕ*).

### 2.3 Model parameters and outputs

As described in the previous sections, the mathematical model is composed of three governing partial differential equations each of which has a number of parameters. These parameters are generally material moduli such as elastic Young's modulus, diffusion coefficient, nutrient consumption rate, overgrowth rate, etc. The total number of parameters (geometrical, material and numerical) exceeds 20. For a more detailed description of the parameters, the readers may refer to [12]. In order to employ an ANN model to the problem, only four of the most influential parameters are chosen to be as the input parameters. Here the shear modulus for the wall matrix (*μ*), shear modulus of collagen fibers (*η*), overgrowth constants ($k_{g}$) and the inflammation rate ($R_{s}$) are the four selected ones. The first and second parameters represent the mechanical resistance while the third and the fourth one account for the underlying biological process. The typical values of parameters appearing in equations (5)-(10) are listed in Table 2. The parameters that are assumed to serve as the input variables of the ANN model are highlighted with asterisks.

**Table 2:** Model and material parameter (the varying parameters serving as ANN inputs are highlighted with asterisks).

Description	Parameter	Value	unit
Blood pressure	*p*_*max*_	1	kPa
*Overgrowth constant	*k*_*g*_	0.25	${\text{Time}}^{-1}$
Cell consumption	*R*_*c*_	10^−3^	$\mu \text{g}.{\mu \text{m}}^{-3}.{\text{Time}}^{-1}$
*Inflammation rate	*R*_*s*_	0.1	$\mu \text{m}.{\text{Time}}^{-1}$
*Wall shear modulus	*μ*	10	kPa
Wall Poisson ratio	*ν*	0.49	–
*Free energy parameter (Fibers shear modulus)	*η*	100	kPa
Free energy parameter	*β*	1	–
Free energy parameter	*ρ*	0.5	–
Phase-field parameter	*ϵ*	1	–
Phase-field parameter	*M*	10	–
Mesh size (coarse/fine)		5/0.5	μm

**Figure 4:**
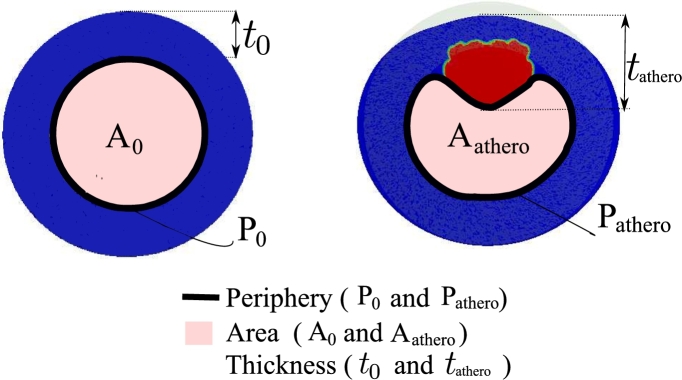
Definition of output parameters of the ANN network for characterizing atherosclerosis in terms of initial (left) and final (right) configuration.

The output of the ANN system should be defined in such a way that characterizes and quantify the atherosclerotic plaque in an artery. To do so, three parameters are introduced as follows (see Fig. 4).

1. Change of lumen cross-section: As a result of stenosis due to the atherosclerotic plaque, the lumen cross-section is reduced. If the cross-section area of the healthy and pathological lumen is called $A_{0}$ and $A_{athero.}$ respectively, the ratio $\frac{A_{0}-A_{athero.}}{A_{0}}$ is an index of artery stenosis.
2. Change of lumen periphery: Similar to the lumen cross-section change, one can define an index for the amount of circumferential extension of the artery wall using the periphery of the lumen cross-section. The periphery of the healthy and atherosclerotic lumen cross-section is denoted by $P_{0}$ and $P_{athero.}$, respectively. Hence, the ratio $\frac{P_{0}-P_{athero.}}{P_{0}}$ reflects the tangential dilatation.
3. Maximum change of wall thickness: Obviously, the maximum change of the wall thickness is an indicator of the atherosclerosis status. The ratio $\frac{t_{0}-t_{athero.}}{t_{0}}$ in which $t_{0}$ and $t_{athero.}$ refer to the healthy and pathological wall thickness showing the degree of thickening.

## 3 Data generation and ANN model

In this section, the data generation process is explained. Also, the details of the ANN model development are given.

### 3.1 Data generation

To generate the database, around 200 multi-physics FEM simulations were automatically generated and solved using a combination of MATLAB (R 2019, MA, US) and ANSYS (V19.2, PA, US). To automatically run the simulations, a MATLAB script was developed first to write ANSYS input files with various materials properties. ANSYS was connected to MATLAB to run the simulations, and the script runs the simulations in batch mode. For more details, the readers are referred to [31], [32].

**Figure 5:**
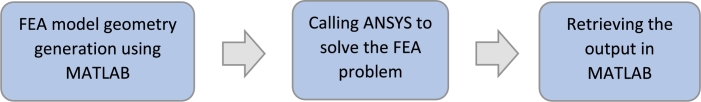
A representation of three main steps in the data generation process.

Except for the four chosen input parameters (shear moduli of artery wall and collagen fibers besides the overgrowth factor and inflammation rate), other model properties, including the geometry, loading, and boundary conditions, were kept unchanged for all simulations. As explained above, the data generation process has three steps as schematically shown in Fig. 5. Each simulation took approximately 2 hours, which results in a total time of 400 hours for generating the whole database.

### 3.2 ANN model

**Figure 6:**
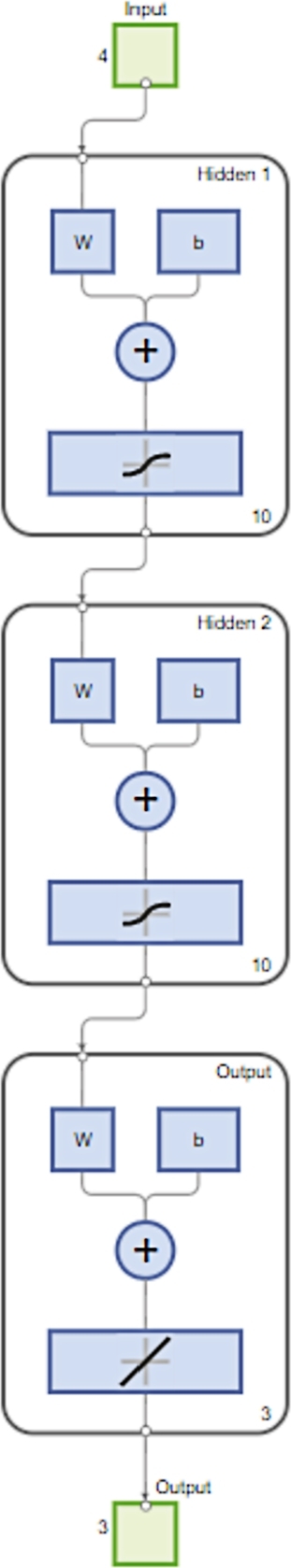
Schematic representation of the ANN model.

**Figure 7:**
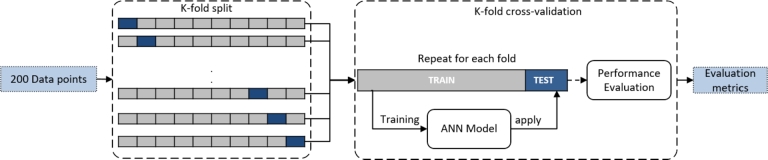
Schematic representation of the cross-fold validation used in this study.

For the regression analysis, a feed-forward network was used with two layers of 10 hidden neurons and a scaled Levenberg-Marquardt (LM) back propagation training function. Initially a very simple network with just a single hidden layer and just a few neurons in the layer was developed. By gradually expanding and examining the network, it was found out that two hidden layers and 10 neurons in each layer are the proper choices of the network for the atherosclerosis problem in this study. A hyperbolic tangent activation function was used for each hidden layer. For the last regression output layer, Linear activation was used. The LM algorithm is specifically designed to minimize the sum-of-square error function. The input layers included 4 features and all 3 outputs are estimated simultaneously. The loss function was calculated based on the mean squared errors of all 3 outputs. A schematic representation of the model is demonstrated in Fig. 6. To evaluate the performance of the neural network, the network was trained and tested on the data set using a 10-fold cross-validation approach rather than a typical training and validation. In the K=10-fold cross-validation used here, we repeated the analysis K=10 times. First, the data set is randomly partitioned into 10 folds. Each time: 9 folds (9/10 of data) are used for training and one fold (1/10 of data) for the test. The same process has been repeated 10 times; a different fold is used for the test each time until all data points are used once as a test point. The evaluation will be the average of all folds at the end. The validation set has not been seen during that training cycle in each interaction. The training was performed with an initial 100 epochs with an early stopping for better generalization and over-fitting prevention. During the training, the error of the validation set was monitored. When the network begins to overfit the data (based on an increase in validation set error), the training is stopped, and the weights and biases at the minimum of the validation error are retrieved. The final performance assessment was done by averaging the performance of all folds. A representative portion of the data set, used in this study, is given in Appendix A. The validation and testing process is also schematically depicted in Fig. 7. The metrics used to quantify the performance for all three parameters were mean squared error (MSE), root-mean-square error (RMSE), and Pearson's correlation coefficient between targets (true values) relative to outputs (predictions). The network architecture and training were implemented in MATLAB 2022a (MathWorks Inc., Natick, Massachusetts), using Deep Learning Toolbox. The training for a single run on a computer equipped with 32 GB of RAM and NVIDIA GTX 1080 ti (11GB) takes 30 seconds while the trained model predicts the parameters for a test input in ∼ 0.3 seconds.

## 4 Results and discussion

In this work the FEM-based numerical tool serves as a virtual data generator, meaning that the problem is solved for different values of input parameters. The output parameters are monitored constantly, and finally, a data set is created to be used in the ANN model. The numerically captured data in this virtual Lab are, indeed, a substitution for experimental/clinical data collected in the actual Lab. Once the ANN model is sufficiently trained, one can examine the accuracy of the predictions made by the ANN model in comparison with the simulation results.

It is obvious that if experimental/clinical data were available, one could readily replace the simulated data with laboratory ones. Thanks to new technologies such as Positron Emission Tomography (PET), see e.g., [33], [34], one can detect atherosclerotic lesions in the arteries to a great extent. However, conducting thousands of experiments on an artery undergoing atherosclerotic overgrowth and monitoring its state over the course of a long time (in the order of months) is not an easy and inexpensive task. The objective here is to investigate how an ANN-based and data-driven model is capable of reproducing numerical results. Undoubtedly, the parameters in the mathematical model can be calibrated using real clinical data. However, here a physically meaningful range is estimated and a sequence of numerous simulations is performed by choosing different values within this range.

**Figure 8:**
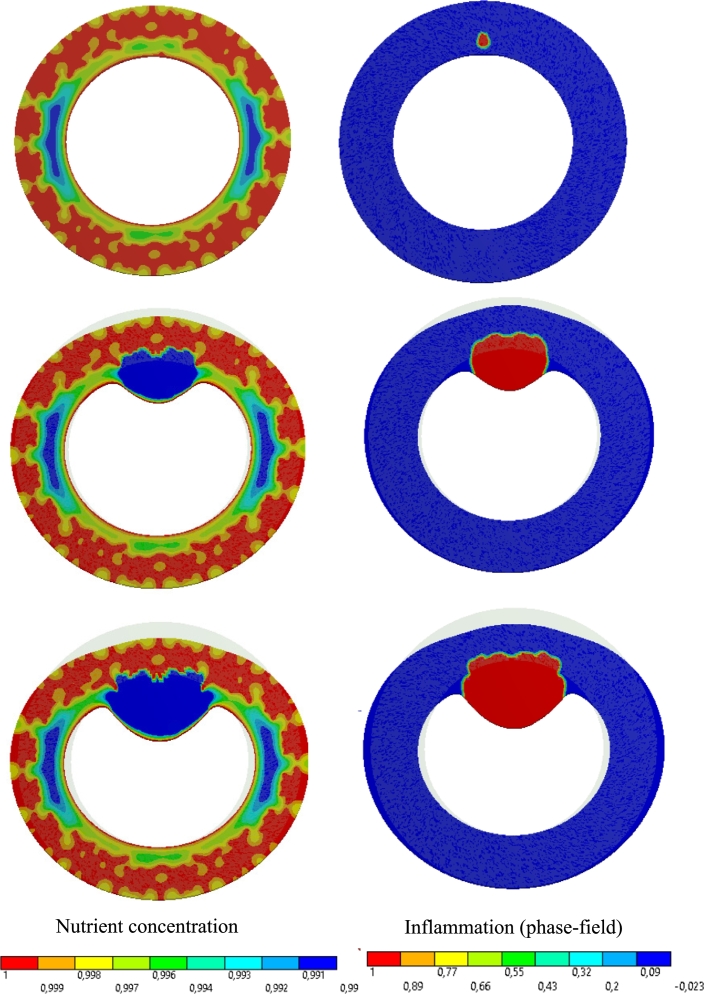
Development of atherosclerotic lesion (right) and its influence on the wall nourishment (left) in the course of the time, from top to bottom.

To provide the readers with a more clear picture of the FEM-based results, the simulation data are presented here for one specific test case. For visualization purposes, a 2D cross-section of the artery is illustrated in Fig. 8. It shows different snapshots of the nutrient distribution in the presence of VVs as well as the inflammatory region. It can be found that how the inflammation leads to the thickening of the artery wall as an indication of atherosclerosis pathology.

**Figure 9:**
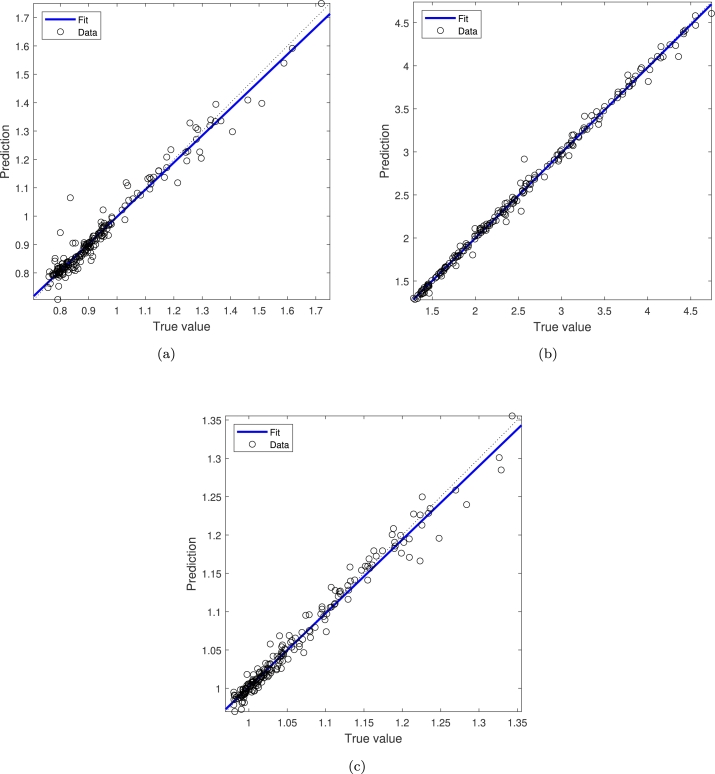
Linear regression of targets (true values) relative to outputs (predictions) for (a) Cross-section, (b) Thickness and (c) Perimeter.

**Figure 10:**
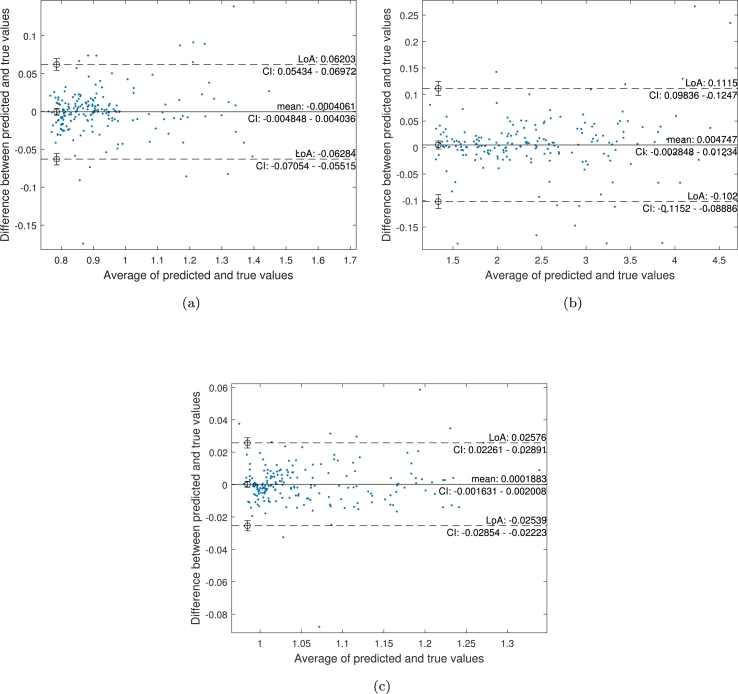
Bland–Altman plots of the agreement between reference values (ground truth) and predicted values for (a) Cross-section, (b) Thickness and (c) Perimeter. The black line shows the mean difference between the predicted and true layer thickness, and the dashed lines show a 95% confidence interval (CI) for the limits of agreement (LoA).

The results obtained from the FE simulations are used to train and validate an ANN model. Results obtained from the trained ANN model (for the three parameters of interest) are shown in Fig. 9 (a-c). The agreement between the predicted cross-section, thickness, and perimeter and the reference ones can be observed in the Bland–Altman plots depicted in Fig. 10 (a-c). Each plot shows the difference between the predicted and true values against the average of them and permits a visual assessment of the distribution of errors. From the observation of these plots, it is worth mentioning that the results of our method are similar to reference values and do not show a substantial bias, as the mean of differences between the values is close to 0. The 95% limits of agreement for thickness estimation are slightly higher than the other two parameters.

It is seen that the ANN model is capable of quick and accurate predictions of atherosclerosis. Table 3 gives the RMSE, MSE and correlation coefficient values from the ANN model for all three parameters. The model provides the opportunity to calculate atherosclerosis-related parameters with the accuracy of high-fidelity FE simulations but with remarkably better computational efficiency. This model gives predictions in a fraction of a second while each of the FEM simulations takes at least 120 minutes. It should be emphasized that the computational gain would be even more remarkable if 3D reference simulations were conducted to generate the required data set. In addition to the input data used to generate the data set, the model can be used for any combination of arbitrary input parameters within the range of training data. It should also be emphasized that compared to the literature and different trained ANN models for a variety of regression tasks, we used a rather small data set (180 training data, and 20 test data).

**Table 3:** Prediction model performance for all 3 parameters.

	RMSE	MSE	Correlation coefficient
Cross-section	0.0342	0.0011	0.9812
Thickness	0.0570	0.0032	0.9979
Perimeter	0.0118	0.0001	0.989

## 5 Conclusions

In this paper, we developed an ANN model for analyzing atherosclerosis in a computationally efficient manner. Multi-physics FEM simulations were used to create the required database. A feed-forward ANN architecture with two hidden layers, each possessing 10 neurons, was used and a scaled Levenberg-Marquardt (LM) backpropagation training function was used for the training process. A rather limited number of data points were used to train and validate the network. The trained network is not only accurate but also remarkably faster than the corresponding FEM simulations. It should however be emphasized that the developed model in this study is the first step towards a more comprehensive multiphysics-based ANN model for the prediction of atherosclerosis.

This work is subject to certain limitations and assumptions which imply possible direction for further development. One can classify further extension to three main paths: The enhancement of the simulations (FEM-model), incorporation of clinical data, and also improvement of ANN architecture. To improve the simulation part, one can improve the arterial constitutive behavior such as the incorporation of fiber-induced anisotropy into the inflammation equation. Currently, the inflammatory response is assumed to be isotropic despite anisotropic elastic response of the artery wall. Besides the improvement of constitutive modeling, a parallelized computational platform can justify moving to a 3D model with a few million of DoFs.

Additionally, quantitative validation and consequently calibration of the FEM model using real clinical data, such as histological imaging of the atherosclerotic arteries, is required. Another interesting direction concerning the usage of clinical data is to look at the inverse problem, meaning that one identifies the input parameters via analyzing the output data instead of vice versa. In practice, the ultimate objective is to replace the numerical output data with early-stage clinical medical images and then estimate the parameters. This is of significant practical importance due to the fact that one can use the identified parameters for prediction purposes such as prognosis of the pathology and finally they can assist the surgeons with decision-making on the required surgical/therapeutic measures. Undoubtedly, follow-up medical images are the touchstone using which one can improve the model's predictive capability.

Regarding the ANN approach, a feed-forward architecture seemed to be a proper choice for the studied problem, and our results confirmed that too. For future extensions of this work, it would be interesting to model the evolution of atherosclerosis and its related parameters in a (pseudo)-time series manner. For that purpose, Recurrent Neural Networks (RNNs), such as Long Short Term Memory (LSTM) and Gated Recurrent Units (GRU), are probably proper choices.

## Funding statement

Meisam Soleimani was supported by 10.13039/501100001659Deutsche Forschungsgemeinschaft [TRR 298].

Mohammad Mirkhalaf was supported by 10.13039/501100000923Australian Research Council [DE210100975].

Mohsen Mirkhalaf was supported by Vetenskapsrådet (10.13039/501100004359Swedish Research Council) [2019-04715].

## CRediT authorship contribution statement

Mohsen Mirkhalaf: Conceived and designed the experiments; Analyzed and interpreted the data; Wrote the paper.

Meisam Soleimani; Behdad Dashtbozorg: Conceived and designed the experiments; Performed the experiments; Analyzed and interpreted the data; Contributed reagents, materials, analysis tools or data; Wrote the paper.

Mohammad Mirkhalaf: Analyzed and interpreted the data; Contributed reagents, materials, analysis tools or data; Wrote the paper.

## Declaration of Competing Interest

The authors declare that they have no known competing financial interests or personal relationships that could have appeared to influence the work reported in this paper.

## Figures and Tables

**Table:** 

No.	Input 1 (*μ*)	Input 2 (*η*)	Input 3 (*k*_*g*_)	Input 4 (*R*_*S*_)	Output 1 (A/A0)		Output 2 (P/P0)		Output 3 (t/t0)	
					FEM	ANN	FEM	ANN	FEM	ANN
1	89236660	839738333	4.2925	6.6318	0.8253	0.8189	2.9628	2.9278	1.0606	1.0625
2	2274556	685016071	2.5439	12.9986	0.7733	0.8034	2.3190	2.3007	1.0218	1.0272
3	27545082	710590398	3.4152	17.5159	1.1749	1.1810	3.4450	3.3772	1.1629	1.1674
4	678269752	50295146930	2.9451	11.9447	0.8058	0.8042	2.5782	2.6030	1.0406	1.0424
5	50941449	264768421	1.0344	12.0709	0.9421	0.9396	1.3583	1.3499	0.9898	0.9957
6	10000000	100000000	2.5000	10.0000	0.8086	0.8144	2.0982	2.0981	1.0195	1.0147
7	59552786	578790724	2.5397	6.3373	0.8903	0.8948	1.8648	1.8620	1.0058	1.0065
8	303195068	29357395923	4.3988	12.9473	1.3466	1.3386	4.2577	4.2808	1.2233	1.2205
9	510210307	40665665948	4.5755	9.1418	1.0376	1.0131	3.8356	3.9574	1.1318	1.1373
10	90297635	386730396	2.5980	13.9589	0.8273	0.8176	2.3694	2.3906	1.0510	1.0361
11	82939475	598613536	2.5726	7.8532	0.8518	0.8482	1.9943	1.9850	1.0145	1.0106
12	76936507	743434369	2.2516	16.4074	0.7904	0.8079	2.2120	2.2144	1.0271	1.0250
13	347610189	350315439	2.0306	17.6133	0.8065	0.8260	1.9949	2.0340	1.0422	1.0186
14	402440215	46241249144	3.0313	8.1144	0.7865	0.7877	2.3364	2.3259	1.0196	1.0127
15	31784213	366022670	1.7682	9.6443	0.9026	0.9034	1.6581	1.6579	0.9990	1.0012
16	97914228	355225813	2.5276	9.5994	0.8205	0.8181	2.0651	2.0697	1.0262	1.0140
17	264529139	28006271326	1.6808	17.3690	0.7944	0.8021	1.8225	1.8609	0.9908	1.0031
18	538708087	40701488244	1.6110	17.3934	0.8073	0.8089	1.7749	1.7745	0.9888	0.9996
19	72731318	692886960	3.3745	19.2550	1.2794	1.2414	3.4998	3.3804	1.2019	1.1813
20	60701329	565923845	2.6142	9.9444	0.8002	0.8082	2.1692	2.1619	1.0242	1.0181

## Data Availability

Data will be made available on request.

## Appendix A

In this Appendix, a small portion of data used for training and validating the ANN model is given. There are 4 inputs and 3 outputs for each data point as given in the following table. The complete data set will be provided to an interested reader upon request.

## Footnotes

1 If the model is extruded in the third dimension (axial direction) and the same size of the mesh is kept, the total number of degrees of freedom exceeds 10^7^ that is computationally prohibitive.
